# Protocols for *Plasmodium* gametocyte production in vitro: an integrative review and analysis

**DOI:** 10.1186/s13071-022-05566-3

**Published:** 2022-12-05

**Authors:** Roukayatou Omorou, Ibrahim Bin Sa’id, Michael Delves, Carlo Severini, Yobouet Ines Kouakou, Anne-Lise Bienvenu, Stephane Picot

**Affiliations:** 1 Malaria Research Unit, UMR 5246 CNRS-INSA-CPE-University Lyon1, University of Lyon, 69100 Villeurbanne, France; 2 Institut Agama Islam Negeri (IAIN) Kediri, 64127 Kota Kediri, Jawa Timur Indonesia; 3 Department of Infection Biology, Faculty of Infectious Tropical Diseases, London School of Hygiene and Tropical Medicine, Keppel Street, London, WC1A 7HT UK; 4 Dipartimento Di Malattie Infettive, Istituto Superiore Di Sanità, Rome, Italy; 5 Service Pharmacie, Groupement Hospitalier Nord, Hospices Civils de Lyon, Lyon, France; 6 Institut de Parasitologie Et Mycologie Médicale, Groupement Hospitalier Nord, Hospices Civils de Lyon, Lyon, France

**Keywords:** *Plasmodium* spp, Malaria, Gametocyte, In vitro, Ex vivo, Protocols, Transmission-blocking

## Abstract

**Background:**

The production of *Plasmodium* gametocytes in vitro is a real challenge. Many protocols have been described, but few have resulted in the production of viable and infectious gametocytes in sufficient quantities to conduct research on—but not limited to—transmission-blocking drug and vaccine development. The aim of this review was to identify and discuss gametocyte production protocols that have been developed over the last two decades.

**Methods:**

We analyzed the original gametocyte production protocols published from 2000 onwards based on a literature search and a thorough review. A systematic review was performed of relevant articles identified in the PubMed, Web of Sciences and ScienceDirect databases.

**Results:**

A total 23 studies on the production of *Plasmodium* gametocytes were identified, 19 involving in vitro* Plasmodium falciparum*, one involving *Plasmodium knowlesi* and three involving ex vivo* Plasmodium vivax*. Of the in vitro studies, 90% used environmental stressors to trigger gametocytogenesis. Mature gametocytemia of up to 4% was reported.

**Conclusions:**

Several biological parameters contribute to an optimal production in vitro of viable and infectious mature gametocytes. The knowledge gained from this systematic review on the molecular mechanisms involved in gametocytogenesis enables reproducible gametocyte protocols with transgenic parasite lines to be set up. This review highlights the need for additional gametocyte production protocols for *Plasmodium* species other than *P. falciparum*.

**Graphical abstract:**

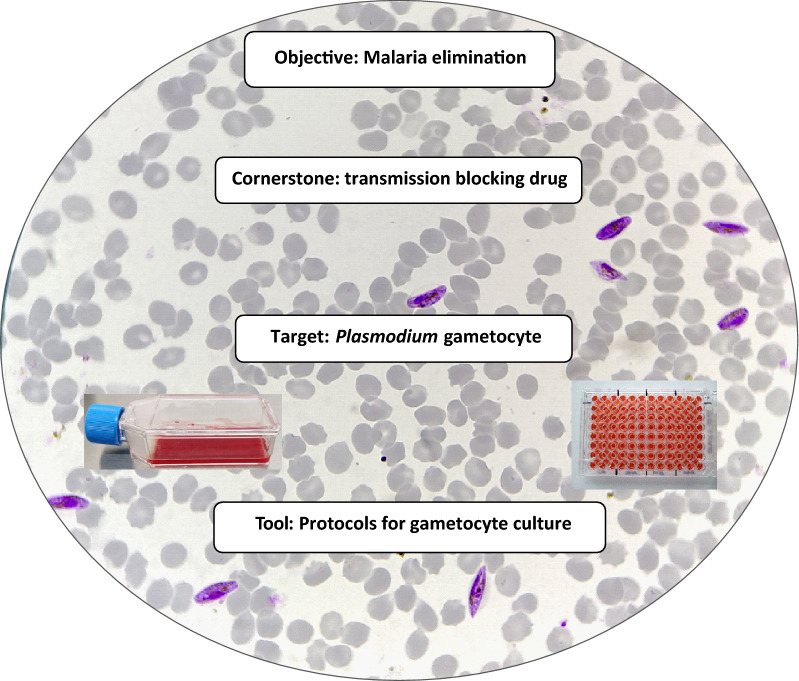

## Background

The massive efforts implemented in recent years to control malaria have avoided approximately 1.7 billion malaria cases and 10.6 million malaria-related deaths worldwide [1]. However, these estimates in the most recent World Malaria Report (2021) are 241 million cases and over 627,00 deaths [2]. A considerable asset in the fight against malaria was provided by the malaria control and elimination strategies that have been implemented, notably the Global technical strategies for malaria 2016–2030 (GTS) adopted in 2015 [3]. The greatest achievements were obtained in symptomatic malaria cases based on the test, treat and track strategy scaled up a decade ago by WHO. More recently, additional attention has been given to asymptomatic malaria, since there is a large body of evidence indicating that asymptomatic parasite carriers have a significant impact on the odds of malaria transmission [4]. Children and adults may harbor sexual forms circulating in the blood for weeks without fever or symptoms, providing an undetected reservoir of malaria parasites. In the path leading toward malaria elimination, this reservoir is a major challenge that needs to be met, particularly given that most of the antimalarial drugs have a limited effect against gametocytes [5, 6]. Although first-line artemisinin-based combination therapies (ACTs) show partial activities against gametocytes [7–9], their effects on mature gametocytes are limited [10, 11]. As a result, the number of studies focusing on gametocytes have increased significantly over the past decade [12]. Research ranging from in-depth understanding of gametocyte biology to the development of drugs, vaccines or diagnostic techniques have a common thread: the need for the reproducible and reliable production of viable gametocytes in vitro.

Gametocytes are the cornerstone of parasite transmission from humans to mosquitoes [13]. The time of their appearance in the peripheral blood after a first erythrocytic cycle is estimated to be 7–15 days for *Plasmodium falciparum*, and shorter for other* Plasmodium* species (1–3 days) [14–17]. There are five (I–V) morphological stages of maturation [18]. The factors associated with gametocyte carriage are complex and not fully understood [19]. Human genetic background, environmental aspects, including climate factors, asexual parasitemia, allelic divergence in specific genes of *Plasmodium* strains, fever or hematopoiesis may all have an impact on sexual differentiation commitment, gametocyte maturation and viability and, ultimately, transmission to mosquitoes [19, 20]. It is not surprising that the successful production of viable gametocytes in vitro may have only some of these factors in common and thus additional factors related to experimental conditions may be required.

In 1976, Trager and Jensen first reported *P. falciparum* continuous culture in human red blood cells (RBCs) [21]. However, while this method allows the occasional appearance of gametocytes, it rarely results in mature and viable gametocytes being obtained. Between the late 1970s and end of the 1990s, various groups of researchers developed protocols to produce sexual stages in vitro [22–28], but reproducibility still remains challenging [29, 30]. The commitment of asexual forms into gametocytes varies widely depending on the *Plasmodium* clone and gametocytogenesis induction method [31, 32]. Studies have shown that the microenvironmental conditions of the parasite, such as high parasitemia, drop in hematocrit, nutrient starvation and lactic acid concentration, are important factors affecting the success of the protocol [33–35]. A genetic analysis indicated that sexual transcripts increased under conditions of high versus low parasitemia [36]. Taken together, these parameters complexified the reproducibility and reliability of gametocyte production in vitro. Extensive protocols were published in 2015, providing a comprehensive approach to the parameters required for routine in vitro culture of *P. falciparum* gametocytes [31, 37].

In this review, we aimed to identify and compare the most recent gametocyte production protocols to favor the dissemination of in vitro tests for drug susceptibility of *P. falciparum* sexual stages. The same approach was used to review protocols for other *Plasmodium* species, although data on the latter remain scarce.

## Methods

An integrative and systematic methodology was used to collect and review published articles on the production of in vitro* Plasmodium* spp. gametocytes. The Preferred Reporting Items of Systematic Review and Meta-Analysis (PRISMA) checklist 2020 (https://prisma-statement.org/) was used for this purpose.

### Eligibility criteria

Studies relevant to the research question were selected according to pre-established criteria. To be eligible, the study had to be published from 2000 onwards, be available and present an original in vitro* Plasmodium* spp*.* gametocyte production protocol. We defined an original protocol as a new method of in vitro gametocyte production that has been developed and tested. Studies that reported ex vivo protocols for *Plasmodium vivax* gametocyte culture were also eligible.

### Search strategy

Our initial approach was to obtain large-scale data on the study subject. To obtain a fairly complete list of research conducted on gametocytes production in vitro, we used the following search terms: “Gametocyte*” AND “Plasmodium”. We used VOSviewer software to generate a term map to analyze the co-occurrence and the relatedness of these two terms, which then allowed us to check the reliability of the query terms. In July 2022, we queried three databases, namely PubMed, Web of Sciences and ScienceDirect, and performed manual searches on the web search engine to gather data. The following query equations were generated: (i) on PubMed/MEDLINE: ((((Gametocyte*) AND (Plasmodium) AND (Gametocyte*[Title/Abstract]) AND (Plasmodium[Title/Abstract])))); filters, from 2000–2022; (ii) on Web of Science (WoS): ((((((ALL = (gametocyte*) AND ALL = (Plasmodium) AND TI = (gametocyte*) AND TI = (Plasmodium) AND AB = (gametocyte*) AND AB = (Plasmodium) AND PY = (2000–2022)))))); (iii) on ScienceDirect: Advanced search, title, abstract and keywords “Gametocyte” AND “Plasmodium”; filters, from 2000 to 2022.

### Study selection, data collection and data analysis

The selection process and data collection were performed by the first author (RO) and checked by two other authors (IBS and YIK). Data from the online query were imported in the reference manager Zotero (version 5.0.96.3). Duplicate articles were eliminated. The first screening was based on title and key words. The first author tehn analyzed the title and abstract of each article resulting from the query so as to select only those articles in which gametocytes were produced in vitro and ex vivo for the *P. vivax* species. Finally, the full text was screened to include studies that met the inclusion criteria.

### Data extraction and management

The first author (RO) screened the full text of included articles to retrieve data of interest. The data collected included information on: gametocytogenesis induction; starting parasitemia; *Plasmodium* strain or clone; synchronous or asynchronous asexual culture; culture medium; adjuvants, N-acetylglucosamine (NAG) or any other similar components used to eliminate asexual forms after induction; duration of the protocol; final gametocytemia count; conversion rate (CR); and viability of gametocytes. The parameters investigated were productivity (quantity of gametocyte produced, CR, viability, protocol duration), reproducibility (number of replicates performed, external/internal validation,) and feasibility (as a qualitative parameter). Excel software (Microsoft Corp., Redmond, WA, USA) was used to generate graphs.

## Results

### Selection process

**Fig. 1:**
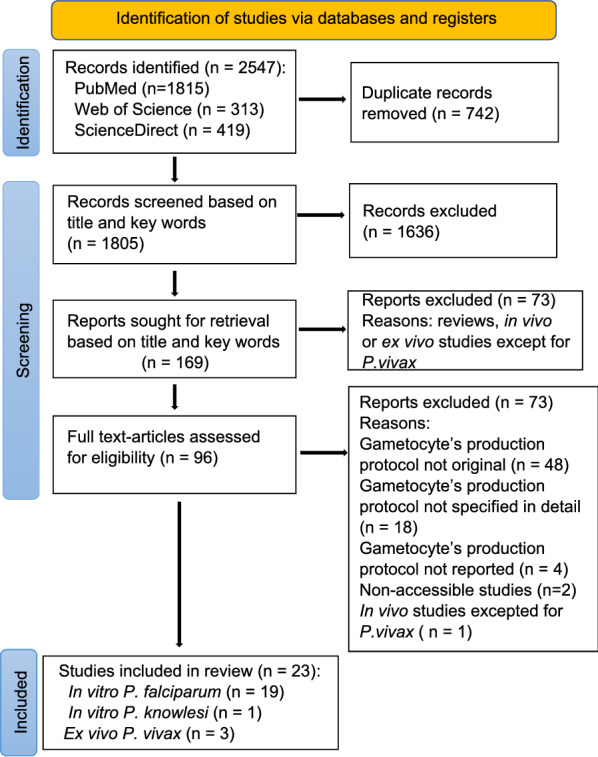
Preferred Reporting Items of Systematic Review and Meta-Analysis (PRISMA) flow diagram of in vitro* Plasmodium* gametocyte production protocols

The online search yielded 2547 results. After the removal of duplicates, 1805 papers were screened for eligibility. First screening of these 1805 papers based on title and key words led to the exclusion of 1636 articles. The 169 remaining studies were screened based on title and abstract; of these, 73 were excluded because they were either in vivo or ex vivo studies (except for *P. vivax*) or reviews. The remaining 96 articles were assessed for inclusion by screening the full text. Ultimately, 23 studies met the inclusion criteria and were included in this review: 19 on *P. falciparum*, three on *P. vivax* and one on *P. knowlesi*. The details of the search process are presented in the PRISMA flowchart shown in Fig. 1. The 23 articles included in this review are listed in Table 1.

**Table 1:** Studies included in the review

Year of publication	First author	Title	Journal	References
2007	Fivelman	Improved synchronous production of *Plasmodium falciparum* gametocytes in vitro	Mol Biochem Parasitol. 154:119–23	[54]
2011	Buchholz	A high-throughput screen targeting malaria transmission stages opens new avenues for drug development	J Infect Dis. 203:1445–53	[29]
2012	Lelièvre	Activity of clinically relevant antimalarial drugs on* Plasmodium falciparum* mature gametocytes in an ATP bioluminescence ‘‘Transmission Blocking’’ Assay	PLoS One. 7:e35019	[39]
2012	Roncalés	Comparison and optimization of different methods for the in vitro production of* Plasmodium falciparum* gametocytes	J Parasitol Res. 2012:927148	[32]
2013	Saliba	Production of *Plasmodium falciparum* gametocytes in vitro	Method Mol Biol. 923:17–25	[48]
2015	Brancucci	An assay to probe *Plasmodium falciparum* growth, transmission stage formation and early gametocyte development	Nat Protoc. 10:1131–42	[41]
2015	Reader	Nowhere to hide interrogating different metabolic parameters of* Plasmodium falciparum* gametocytes in a transmission blocking drug discovery pipeline towards malaria elimination	Malar J. 14:213	[31]
2015	Vera	Purification methodology for viable and infective *Plasmodium vivax* gametocytes that is compatible with transmission-blocking assays	Antimicrob Agents Chemother. 59:6638–41	[51]
2016	Delvesl	Routine in vitro culture of *P. falciparum* gametocytes to evaluate novel transmission-blocking interventions	Nature Protocols. 11:1668–80	[37]
2016	Duffy	Large-scale production of *Plasmodium falciparum* gametocytes for malaria drug discovery	Nat Protoc. 11:976–92	[43]
2017	Demanga	The development of sexual stage malaria gametocytes in a wave bioreactor	Parasit Vectors. 10:216	[38]
2018	Armisteadl	Infection of mosquitoes from in vitro cultivated *Plasmodium knowlesi* H strain	Int J Parasitol. 48:601–10	[47]
2018	Pathak	Cryogenically preserved RBCs support gametocytogenesis of *Plasmodium falciparum* in vitro and gametogenesis in mosquitoes	Malar J. 17:457	[68]
2018	Rangel	Enhanced ex vivo* Plasmodium vivax* intraerythrocytic enrichment and maturation for rapid and sensitive parasite growth assays	Antimicrob Agents Chemother. 62:e02519-17	[52]
2019	Tanakal	Polyunsaturated fatty acids promote Plasmodium falciparum gametocytogenesis	Biol Open. 8:bio042259	[55]
2020	Portugaliza	Artemisinin exposure at the ring or trophozoite stage impacts* Plasmodium falciparum *sexual conversion differently	Elife. 9: e60058	[42]
2020	West	Lactic acid supplementation increases quantity and quality of gametocytes in *Plasmodium falciparum* culture	Infect Immun. 89: e00635-20	[50]
2020	Llorà-Batlle	Conditional expression of PfAP2-G for controlled massive sexual conversion in* Plasmodium falciparum*	Sci Adv. 6:eaaz5057	[46]
2021	Wadi	Investigation of factors affecting the production of *P. falciparum* gametocytes in an Indian isolate	3 Biotech. 11:55	[30]
2021	Boltryk	CRISPR/Cas9-engineered inducible gametocyte producer lines as a valuable tool for* Plasmodium falciparum* malaria transmission research	Nat Commun. 12:4806	[45]
2021	Ridgway	Sex-specific separation of* Plasmodium falciparum* gametocyte populations	Bio Protoc. 11:e4045	[44]
2022	Ramos	Viability and infectivity of *Plasmodium vivax* gametocytes in short-term culture	Front Cell Infect Microbiol. 11:676276	[53]
2022	Dinko	Generation of* Plasmodium falciparum* gametocytes in vitro with specific consideration for field isolates	Method Mol Biol. 2470:121–32	[40]

### Characteristics of the studies

The gametocyte production protocols that were developed in the studies listed in Table 1 are presented here so that they can be produced by others. Most of the studies (96%, 22/23) were performed within the last 10 years. Relevant parameters of the review protocols of in vitro gametocyte production are summarized in Table 2.

**Table 2:** Characteristics of the included studies

References	*Plasmodium* strain/clone^a^	Starting parasitemia (%)	Starting hematocrit (%)	Medium (%)	Type of human serum	NAG	Synchronicity	Induction principle^b^	CR (%)	Mature gametocyte (%)	Viability	Infectivity
[54]	3D7A	2	5	10 HS/5 HS + 0.25 Alb	Pooled AB+	Yes	Sorbitol	Stress (1, 2)	10–30	NA	Yes	Yes
[29]	P2G12 164/GFP	1	1	1 Alb	NA	No	Sorbitol percoll	Stress (1)	NA	NA	Not tested	Not tested
[39]	3D7A, NF54, W2, Dd2, 3D7HT-GFP	0.2	12	15 Alb, then 10 Alb	NA	Yes	Sorbitol	Stress (1, 3)	NA	1–2	Yes	Not tested
[32]	3D7, NF54, Dd2, FCR3, W2, HB3	0.75	2	10 HS	Pooled AB+	No	Sorbitol	Stress (4)	11–23	~ 1.2	Not tested	Not tested
[48]	3D7A, NF54	3–5	4	10 HS	O+	Yes	Sorbitol percoll/No	Stress (1)	NA	NA	Not tested	Not tested
[41]	Pf2004/164-tdTom	0.3	2.5	10 HS	NA	No	Sorbitol	Stress (1, 2)	14–24	NA	Not tested	Not tested
[31]	3D7, NF54, FCR3, W2, HB3, 7G8	0.5	6	0.5 Alb	NA	Yes	No	Stress (1, 3)	~ 33	4	Yes	Not tested
[37]	NF54	1	4	10 HS	Pooled A+	No	No	Stress	NA	~ 2.5	Yes	Yes
[43]	NF54^pfs16–LUC−GFP^	3	5	5 HS + 0.25 Alb	AB+	Yes	Sorbitol MACS	Stress (1)	11–33	–	Not tested	Not tested
[38]	3D7, FCR3	0.8–1.1	6	10 HS	NA	Yes	Sorbitol	Stress (1)	NA	~ 0,1–0.3	Yes	Yes
[68]	NF54	0.6–1	5	10 HS	Pooled A+	No	No	Stress	NA	NA	Yes	Yes
[55]	NF54, 3D7	0.1	6	10 HS/0.5 Alb + PUFAs	A+ /O+	Yes	No	Stress (1)	NA	~ 1	Not tested	Not tested
[42]	NF54-gexp02-Tom, E5-gexp02-Tom, and NF54-10.3-Tom	1.5	3	0.5 Alb + 2 mM choline/10 HS	NA	Yes	Sorbitol percoll	Stress, 5 nM DHA for 3 h	40	NA	Yes	Yes
[50]	NF54	0.5	4	10 HS	O+	Yes	No	Stress + lactic acid	NA	3	Not tested	Yes
[46]	E5-PfAP2-G-DD	NA	4	0.5 Alb/10 HS	NA	Yes	Sorbitol percoll	Condition	90	NA	No	No
[30]	RKL-9 (Rourkela, Odisha)	0.5	10	10 HS	AB+	Yes	Sorbitol	Stress (1)	NA	NA	Not tested	Not tested
[45]	NF54/iGP1 GDV1-GFP-DD	1.5	5	10 HS	AB+	Yes	Sorbitol	Condition	75	4	Yes	Yes
[44]	3D7-gACBG2-GFP	3 – 5	4	~ 2 HS + 0.3 Alb	Pooled O+	Yes	Sorbitol MACS	Stress	NA	NA	Not tested	Not tested
[40]	*Pf* field isolates	2–3	3	10 HS + 0.5 Alb	Pooled O+	Yes	Sorbitol MACS	Stress (2)	NA	NA	Yes	Not tested
[47]	*P. knowlesi* PkH/FZ8	1	0.2 /2	10% Rhesus pooled sera		Yes	Sorbitol percoll	Stress (1)	NA	< 0.05	Not tested	Yes

The “Protocol-type” articles and some studies did not report quantitative gametocyte data. In these articles, the authors state that the methods were validated either by viability assays or by quantitative measurements

*Alb* Albumax II, *CR* conversion rate,* DHA* dihydroartemisin* HS* human serum, *NA* not available, *NAG* N-acetylglucosamine, *PUFAs* polyunsaturated fatty acids,* Sorbitol MACS* MacConkey Agar with Sorbitol

^**a**^For studies that use multiple *P. falciparum* strains, if the authors specify, the gametocytemia and CR reported correspond to the underlined strain

^**b**^All stress-based gametocytogenesis induction uses high parasitemia as a stress factor. Code: (1) drop in hematocrit; (2) parasite conditioned medium; (3) nutrient starvation; (4) lysed uninfected red blood cells

#### *Plasmodium *parasites

In the studies included in this review, the NF54 strain and the 3D7 clone were the most commonly used strains for in vitro experiments with *P. falciparum* (53%, 10/19) followed by the FCR3 [38] and W2 [39] clones. Parasites adapted from clinical isolates were rarely tested [30, 40]. Transgenic lines derived from the NF54 and 3D7 clones were also used to produce gametocytes. Such transgenic lines contain a fluorescent protein that is under the control of the expression of a gametocyte-specific protein leading to accurate and easy-to-perform quantification of gametocytes. These include *Pf*2004/164-tdTom [41], *NF54-gexp02-Tom*, *E5-gexp02-Tom* and *NF54-10.3-Tom* [42]. All of these parasite lines expressed the fluorescent reporter tdTomato under the control of the promoter of the sexual stage-specific gene* gexp02* (PF3D71102500). The 164/GFP P2G12 line [29], NF54-pfs16-LUC-GFP line [43], 3D7HT-GFP [39] and 3D7gACBG2-GFP [44] expressed the fluorescent reporter green fluorescent protein (GFP*).* Inducible gametocytogenesis lines have also been developed. E5 gametocyte-inducible line (E5ind line) is a subclone of 3D7 that contains conditional activation construct AP2-G-DD. Even though this line supported a high production of gametocytes, the gametocytes were not infectious to mosquitoes. *Plasmodium falciparum* NF54 clone was genetically modified with a conditional activation construct GDV1-GFP-DD (*Pf*NF54/iGP1) [45, 46]. The reported *P. falciparum* transgenic lines are summarized in Table 3. Three studies showed data on *P. vivax *ex vivo gametocyte culture, and one study was using *Plasmodium knowlesi* for in vitro gametocyte production. This latter study showed for the first time that infectious gametocytes of *P. knowlesi* could be produced in vitro with a highly synchronized culture, although the production was low.

**Table 3:** *Plasmodium falciparum* transgenic lines reported to date

	Gametocyte fluorescent lines		Gametocyte sex-specific fluorescent lines	Gametocytes inducible lines
	tdTomato fluorescent reporter	GFP fluorescent reporter		
			3D7 gABCG2-GFP [44]	E5-pfAP2-/G-DD [46]
*Pf* transgenic lines	*Pf*2004/164.tdTom [41] NF54-GgexpO2.Tom [42] E5-gexpO2.Tom [42] NF54-10.3-GFP [42]	P2G12_164/GFP [29] NF54.pfs16-Luc-GFP [43] 3D7HT-GFP [39]		NF54/iGP1 GDV1-GFP-DD [45]

*GFP* Green fluorescent protein,* Pf* *Plasmodium falciparum*

#### Synchronization

**Fig. 2:**
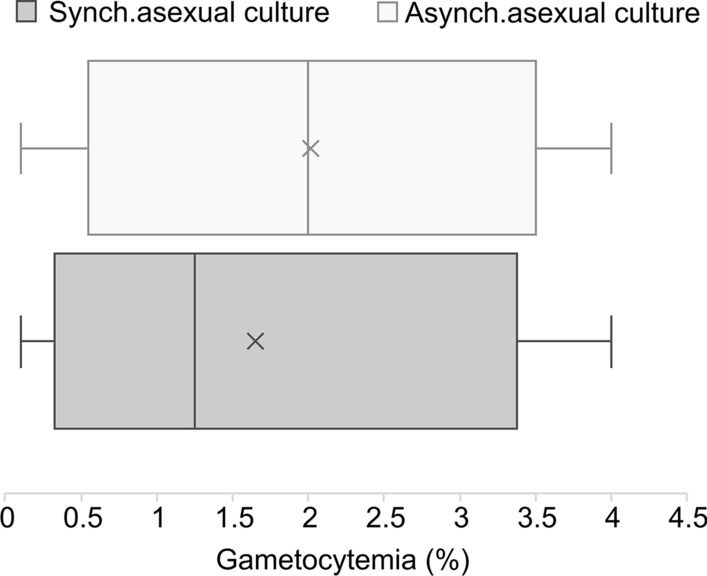
Gametocytemia range based on the asexual parasite synchronicity at the beginning of gametocyte production culture. Mature gametocytes were counted microscopically by performing a Giemsa-stained thin blood smear. Note that 50% of the gametocytemia values are within the box. The vertical line in the box represents the median gametocytemia value, which is about 1.3% and 2% for synchronous and asynchronous culture respectively. The “X” is the mean gametocytemia reported for each variable. The whiskers represent the lower and upper 25% of gametocytemia values. * Asynch.* Asynchronous,* Synch.* synchronous

For most of the protocols, asexual parasites were synchronized or purified at a late maturation stage (15/20 protocols) at the beginning of the experiments. Sorbitol treatment was used in 7/15 studies, while other methods were successfully tested: schizont enrichment by flotation in 1% gelatin [38], both sorbitol treatment and Percoll density gradient [29, 42, 46–48], magnetic columns (MACS) based on paramagnetic propriety of hemozoin [49] or a combination of sorbitol + MACS [40, 43, 44]. Both synchronous and asynchronous culture support gametocytogenesis (Fig. 2). Gametocytemia refers to the quantity of mature gametocytes produced, and some of the gametocytes in the studies were assessed for viability and infectivity (Table 2). Of the five protocols that used asynchronous culture, gametocytemia was between 2.5% and 4% [31, 32, 37, 50]. Demanga et al. [38] obtained a production of mature gametocytes of between 0.1% and 0.3% for both synchronous and asynchronous culture. Synchronous cultures (15/20 protocols) showed gametocytemia ranging from 1% to 3%, with a CR ranging from 11 to 40% [42]. Conditional gametocytogenesis induction provided 4% gametocytemia with 75% CR [41, 43].

*Plasmodium vivax *in vitro continuous cultures have not been achieved so far. As an alternative, ex vivo cultures of gametocytes have been set up by optimizing the enrichment method of clinical isolates and culture conditions. Gametocytes from *P. vivax* clinical isolates were successfully cultured for 48 h after being purified using a Percoll gradient, KCl Percoll gradient or MACS after leukodepletion [51–53]. The addition of KCl to the Percoll gradient was thought to minimize dehydration of cells applied to the gradient [52]. However, Percoll gradient enrichment seemed to preserve parasite viability compared to MACS, with MACS showing higher enrichment. The use of Iscove’s modified Dulbecco’s medium (IMDM) culture medium improved parasite survival [52]. When compared to Roswell Park Memorial Institute medium (RPMI)-1640, IMDM is more concentrated in amino acids, vitamins, glucose and some inorganic salts.

#### Hematocrit

The hematocrit is one of the parameters that widely varies across protocols. The hematocrit at culture initiation was reported by various authors to be 12% [39], 6% (3/20), 5% (4/20), 4% (6/20), 3% (3/20), 2% [32] and 0.2% [47]. A rapid decrease in hematocrit was used as a stress-based induction principle, leading to a reduction from 12% to 6% [39], from 6% to 3% (2/20), then 1.25% for 1 day; subsequent reductions to 2.5% [43], 2% [29] have been reported. As well as hematocrit, cultures had parasitemia ranging from 0.1% to 0.5% (7/20) and from 0.6% to 1% (6/20) and to > 1% (6/20).

#### Culture medium

The basic culture medium most commonly reported for use in in vitro protocols was RPMI-1640 + HEPES + sodium bicarbonate + L-glutamine/or glucose and often an antibiotic (gentamicin). Studies reported the addition to this basic medium of either 10% human serum or AlbuMax II (1% or 0.5% or 15%; Thermo Fisher Scientific, Waltham, MA, USA) [29, 31, 38, 39] or a mixture of serum (5%) + AlbuMax II (0.25%) [29, 43, 44, 54, 55] or a mixture of serum (10%) + AlbuMax II (0.5%) [40]. AB+ and O+ pooled human sera were the most reported blood type used, followed by A+. IMDM + 10% human AB+ serum was used for ex vivo* P. vivax* culture [52, 53]. Culture in the presence of 10% human serum is commonly used, and the corresponding mature gametocytemia ranges from 0.1% to 3% with stress-based induction and is 4% with conditional gametocytogenesis induction. When gametocytes were produced in the presence of AlbuMax II, Reader et al. [31] (0.5% Albumax II) and Lelièvre et al. [39] (10% Albumax II) reported 4% and 1-2% gametocytogenesis, respectively, when using stress-based induction. Serum +AlbuMax II mixture (5% human serum + 0.25% AlbuMax II) has been used to achieve CR of 11–33% [43]. Incubation conditions of cultures were almost similar for all of these studies.

#### Productivity of gametocytes

We screened the number and viability of gametocytes produced to assess protocols’ efficiency. Gametocytemia is usually calculated as the number of gametocytes per 100 RBCs. The CR relates to the proportion of ring forms that lead to gametocytes after their induction (but does not report on whether the gametocytes are capable of continued development to maturity). The CR is estimated as the number of stage II gametocytes that appear in culture 2 days after the appearance of the ring forms. The CR can thus be expressed by the following formula [23]:

$$Conversion rate=\frac{\mathrm{no}. \, \mathrm{of } \, \mathrm{stage II } \, \mathrm{gametocytes } \, \mathrm{per }100 \, \mathrm{RBCs } \, \mathrm{counted } \, 48\mathrm{ h } \, \mathrm{after } \, \mathrm{ring } \, \mathrm{forms}}{\mathrm{no}. \, \mathrm{of } \, \mathrm{ring } \, \mathrm{forms } \, \mathrm{per }100 \, \mathrm{RBCs } \, \mathrm{on } \, \mathrm{day }0 \, \mathrm{prior } \, \mathrm{to stage II } \, \mathrm{gametocytes}} \times 100$$

We found that the time required for *P. falciparum* gametocyte production in vitro was approximately equal for all protocols, ranging from 12 to 16 days with an average of 14 days.

For *P. vivax,* the productivity is relative to the rate of gametocyte purification or enrichment. The KCl Percoll density enrichment method provided an average enrichment about 50-fold of parasites (asexual + sexual forms) [52], while the Percoll 60 purification method yielded an average (± standard deviation) of 81.6% (± 30.50%) and the Percoll 45 method resulted in an average of 44.15% (± 33.88%) recovery of gametocytes from clinical isolates [51]. *Plasmodium vivax *ex vivo parasites were cultured for 48 h [52].

#### Induction of gametocytogenesis

The production of gametocytes in vitro requires induction factors, which are either molecular or environmental. Most of the included studies (18/20) used environmental stress factors to trigger gametocytogenesis. The most frequently mentioned natural factors were high parasitemia (all included studies), sudden drop in hematocrit [29–31, 39, 43, 55], conditioned medium from parasites’ culture that contained metabolites and secreted stress factors [29, 40, 43, 54] and nutrient starvation [31]. Adjuvants added were lactic acid [50], fatty acid [55], chloroquine and dihydroartemisin (DHA) [42]. Two studies reported a conditional gametocytogenesis induction principle using a transgenic gametocyte-inducible line. The expression of a gene involved in gametocytogenesis is conditioned by an external factor. The presence or absence of this factor will induce gametocytogenesis and thus gametocyte production in vitro. Llorà-Batlle et al. [46] developed a conditional expression of the *Pf* AP2-G transcription factor while Boltryk et al. [45] established a conditional overexpression of the sexual commitment factor GDV1. GDV1 and *Pf* AP2-G are nuclear proteins known to be master regulators of sexual commitment [45, 46].

#### Gametocytemia and gametocyte viability

The standard method for determining parasitemia is microscopy and involves counting gametocytes on Giemsa-stained smears. However, other techniques, such as flow cytometry [29, 41, 42] and fluorescence microscopy, have been reported. Lelièvre et al. [39] used the Neubauer chamber to count gametocytes following the purification process. The viability of gametocytes is assessed by using the exflagellation assay of mature male gametocytes (gametogenesis induction). While only viable gametocytes can be converted into gametes, it may be that not all gametocytes are infectious. To assess the infectivity of gametocytes, the standard membrane feeding (SMFA) test is used. SMFA involves feeding mosquitoes with a mature gametocyte culture and then evaluating the production of oocysts in the mosquitoes. Only infectious gametocytes will produce oocysts [56]. The following factors promote the induction of gametogenesis: a decrease in temperature, an increase in pH and the presence of the mosquito factor xanthurenic acid [57]. While a female gametocyte develops into a single macrogamete, one male *P. falciparum* gametocyte generates eight flagellated and motile microgametes [58]. Among the reported protocols, only one did not yield viable gametocytes [46] (Table 2).

## Discussion

In the present study, we reviewed and systematically analyzed recently optimized gametocyte production protocols. Nineteen articles on in vitro gametocyte production for *P. falciparum,* published between 2000 and 2022*,* three articles on *P. vivax* and one articles on *P. knowlesi,* were included in this systematic review. Very little data were available for *P. vivax* due to the lack of standardized protocols for easy long-term cultures which precludes the possibility of producing significant amounts of gametocytes for in vitro tests. *Plasmodium vivax* gametocytes were obtained ex vivo for one cycle of maturation after Percoll density or MACS enrichment of clinical isolates. Only one paper reported the production of *P. knowlesi* sexual forms leading to a low number of gametocytes (0.05%) [47]. Importantly, these gametocytes were infectious to mosquitoes and generated oocysts with a low reproducibility rate (~ 50%) [47].

The in vitro production of viable gametocytes is a complex and multifactorial process, but the complexity of gametocytogenesis in vivo is probably much greater, with many host factors that could influence the sexual CR [59]. It is not a straightforward given that the multiple factors affecting in vivo gametocytogenesis could be the same as those required for in vitro gametocyte induction and survival. The analysis presented was focused only on in vitro factors. Many factors favoring gametocytes culture were identified, including the propensity of the parasite for gametocytogenesis, the biological conditions of culture (parasitemia, hematocrit, medium and synchronization) and the factors triggering the induction of the sexual commitment.

Several *P. falciparum* clones/strains are known for their propensity to produce gametocytes in vitro, and the NF54 strain is representative of these culture-adapted parasites [32, 37]. Nevertheless, it is well-known that clones lose their ability to produce gametocytes as the number of in vitro passages increase, probably related to the deletion of genes or the repression of promoters necessary for sexual development, allowing them to outcompete the gametocyte-competent clones in the population. The number of freeze–thaw and asexual cycles before sexual commitment in the studies reviewed here is almost impossible to determine, but it may be a confounding factor to keep in mind for comparison between methods. To overcome these obstacles related to clones and to increase the production of gametocytes, transgenic lines containing fluorescent proteins under the control of the promoter of the sexual stage-specific gene have been used, allowing the development of easy-to-perform gametocyte readout for drug screening assays [29, 39, 41–43]. High CRs of 90% [46] and 75% [45] were achieved with the conditional gametocytogenesis induction principle compared to 10–40% using the stress-based induction principle. However, the protocol that reported a CR of 90% produced gametocytes that were non-infectious to mosquitoes due to a defect in exflagellation.

The biological conditions for in vitro culture have a significant impact on the CR and the harvest of viable stage V gametocytes. While the basic medium is almost always the same, it may be supplemented with different factors at various concentrations, leading to important differences in the final result.

Supplementation of the culture medium with either human serum [46, 60], AlbuMax II [29, 31, 39] or a combination of both in different proportions [40, 43, 54] was reported in the included studies. Supplementation with 10% human serum supplementation is commonly used to produce gametocytes in vitro. It has been speculated that post-2012 batches of Albumax II would no longer be suitable for use as a complete serum substitute because a reduced growth of asexual parasites was observed with these batches. This reduced growth would be expected to result from a modification in the lipid composition of AlbuMax II [43]. Supplementation with 10% human serum may provide a high gametocyte production but may be subject to the huge differences among serum samples depending on the donor’s factors. AlbuMax II is a cheaper but less effective serum substitute, and the cornerstone of this discrepancy may be linked to lipid composition and concentration. Gametocyte maturation may require less lipid than asexual forms. However, the requirement for specific lipids is higher during gametocyte maturation [55, 60, 61]. A comparative study of the lipid composition of a basic medium supplemented with 0.5% AlbuMax II versus 10% human serum showed that AlbuMax II contains less lipids [55], mainly polyunsaturated fatty acids (PUFAs). About 1.5- to twofold more gametocytes were produced using 10% human serum, compared to 0.5% AlbuMax II. This difference was corrected when the medium with AlbMax II was supplemented with PUFAs [55]. The protocols that we reviewed here showed a similar range of gametocytemia across the three categories of basic medium supplementation.

The reported starting parasitemia was different across protocols, probably due to culture conditions and, importantly, the growth profile, which is strain specific. The starting parasitemia must be adjusted according to the infectivity rate of parasites so as to reduce the time required to obtain the parasitemia necessary for the production of gametocytes. Indeed, the longer the culture duration, the greater the reduction in the quantity of mature gametocytes, in part due to the lysis of RBCs. RBCs have a limited life span and are the cornerstone of the maturation of the gametocyte they host. The most recently collected RBCs at the beginning of the culture would thus be important to consider. It is therefore advisable that RBCs for the first day of induction be between 3 and 10 days post-collection [43, 54]. Storage of RBCs at 4 °C for up to 2 weeks does not appear to hinder the success of gametocyte culture [37].

Synchronous or asynchronous asexual culture allows gametocyte production with no significant difference in productivity (Table 2). However, synchronous gametocyte cultures are important for performing certain parasite stage-dependent studies in proteomics, transcriptomics and drug screening.

It is also important to consider the cost, duration and practicability of the selected protocol. A protocol with several synchronization steps using a culture medium supplemented with human serum will be more time-consuming and costly than a protocol with an asynchronous culture in the presence of the AlbuMax II/serum. Therefore, protocols should be carefully chosen according to the experiments to be performed with gametocytes, and probably according to the skills and facilities, with the ultimate goal to produce sufficient viable and infective gametocytes.

Based on evolutionary theory, it has been suggested that the parasite differentiates into sexual forms when it detects stress factors in its environment [62]. It has also been considered that a subpopulation of circulating parasites commits to sexual differentiation at each cycle, thereby providing a continuous supply of gametocytes for transmission through mosquito bites [19]. This latter option probably occurs more frequently in areas of peri-annual transmission where there are frequent mosquito’s bites. While significant progress has been made in the comprehension of sexual conversion in vivo, it is still impossible to predict the occurrence of gametocytogenesis during malaria infection [59]. The induction of the molecular mechanism leading to the expression of the transcriptional factor *Pf*AP2-G in asexual parasites is a critical triggering step to produce gametocytes [63–66]. It is suggested that *Pf*ap2-g is mainly expressed at the ring and late schizont stages [67]. Thus, gametocytes differentiation can occur in the same or next replication cycle according to the level of AP2-G protein [63, 67]. Stress-based induction by environmental factors was used in the majority of studies involving *P. falciparum* (18/20). These factors include high parasitemia, drop in hematocrit, nutrient starvation, addition of parasite-conditioned medium, lactic acid or drugs. Conditional induction was used with the two reported *P. falciparum* gametocyte-inducible transgenic lines.

Whether it is the gametocyte count or the CR, these two methods of calculation would present a bias. The CR is calculated as the ratio of gametocytes compared to the number of rings counted 48 h before. However, some earlier gametocytes will not mature, resulting in a lower number of mature gametocytes at the end of culture. Conversely, gametocytemia is expressed as the number of gametocytes at the end of culture compared to the number of RBCs (number of gametocyte/number of RBC). In this case, the issue of RBC lysis occurs throughout the production period, as the RBCs are consumed in asexual cycles and also age and become more fragile. Thus, when gametocytes reach maturity, there are far fewer RBCs than earlier in the culture, and this tends to lead to an overestimation of the real gametocyte number in the culture. Therefore, both methods would overestimate the quantity of gametocytes produced. An alternative would be to estimate the quantity of gametocytes as the number of gametocytes per microliter of culture medium. This would dispense with variables and also provide an accurate measurement of the quantity of gametocyte biomass produced.

## Conclusions

Several biological parameters contribute to a more optimal production of viable and infectious mature gametocytes. Insights into the molecular mechanisms involved in gametocytogenesis have made it possible to produce gametocytes with transgenic parasite lines. These protocols would appear to be more reliable and robust. However, the question of viability and infectivity of these gametocytes remains to be studied. It is worth highlighting that extensive efforts have been made to develop reliable methods to produce gametocytes in vitro and ex vivo which would in turn allow further studies aimed at better understanding this life stage of *Plasmodium* and developing specific tools, such as malaria transmission-blocking drugs and vaccines. Furthermore, there is a need for additional gametocyte production protocols for non-falciparum *Plasmodium* species. A joint effort should be made by the community of scientists involved in malaria control studies to standardize *Plasmodium* cultures for gametocytes; this will represent a real turning point in bridging the knowledge gap on the biology of all plasmodia.

## Data Availability

All data are available upon reasonable request.

## Abbreviations

ACTs: Artemisinin-based combination therapies
CR: Conversion rate
DHA: Dihydroartemisin
GFP: Green fluorescence protein
GTS: Global technical strategies for malaria
IMDM: Incomplete Iscove’s modified Dulbecco’s medium
MACS: Magnetic columns
NAG: N-acetylglucosamine
PRISMA: Preferred reporting items of systematic review and meta-analysis
PUFAs: Polyunsaturated fatty acids
RBCs: Red blood cells
RPMI: Roswell Park Memorial Institute medium
SMFA: Standard membrane feeding assay
